# A Small Cut, a Big Consequence: A Case Report of a Radial Artery Pseudoaneurysm

**DOI:** 10.7759/cureus.105689

**Published:** 2026-03-23

**Authors:** Sachin D Potdar, Muzna M Al Sawafi

**Affiliations:** 1 Emergency Medicine, Medical City for Military and Security Services, Muscat, OMN

**Keywords:** arterial aneurysms, emergency medicine physician, emergency vascular injuries, minor injuries, surgical emergencies

## Abstract

Radial artery pseudoaneurysm is an uncommon complication of penetrating wrist trauma, more frequently associated with iatrogenic procedures than with trivial lacerations. While radial artery injuries from penetrating trauma can present as isolated vessel damage, they frequently involve complex injuries affecting adjacent structures, such as veins, tendons, and nerves.

This case report highlights a rare instance of an isolated radial artery pseudoaneurysm resulting from a minor laceration, emphasizing the need for heightened clinical suspicion, even in seemingly innocuous injuries. We report the case of a middle-aged male who sustained a knife-induced volar wrist laceration less than 1 cm in length. Computer tomography (CT) angiography at presentation excluded major vascular injury, and the wound was sutured. Ten days later, the patient returned with a pulsatile swelling at the wound site. Doppler ultrasonography confirmed a radial artery pseudoaneurysm with partial thrombus formation. The patient underwent vascular surgical repair, with a good outcome. This case highlights the importance of thorough vascular assessment in apparently minor wrist injuries. Incorporating bedside ultrasonography in the emergency department (ED) may support earlier recognition of vascular complications.

## Introduction

Peripheral arterial injuries commonly present to the emergency department (ED), but management is often focused on controlling bleeding and wound closure, particularly in busy clinical environments. Pseudoaneurysm of the radial artery is rare and usually reported following arterial cannulation or other iatrogenic causes. Although penetrating trauma to the radial artery can sometimes result in isolated vessel damage, it often leads to more complex injuries that also involve nearby structures, such as veins, tendons, and nerves [1]. Traumatic pseudoaneurysms after small lacerations are infrequently described. We present a case of a radial artery pseudoaneurysm that developed after a seemingly trivial wrist injury, highlighting the need for vigilance and follow-up in such cases [2,3]. This case underscores that while trans-radial access for cardiac procedures is a common cause of vascular complications, like pseudoaneurysms, even minor traumatic lacerations warrant careful evaluation for similar sequelae [4].

## Case presentation

A 40-year-old male presented to the ED with a knife-induced laceration to the volar aspect of his left wrist, sustained while cutting meat. The wound measured about 6 mm and was initially spurting blood, but it stopped with pressure. On arrival, he had stable vital signs and no active bleeding. Neurovascular examination of the left hand was intact.

Given the anatomical location of the wound and the initial nature of the bleeding, computer tomography (CT) angiography was performed. It demonstrated intact arteries of the left upper limb, with no contrast extravasation at the site of injury, and revealed no additional bony, tendon, or muscle injury.

**Figure 1:**
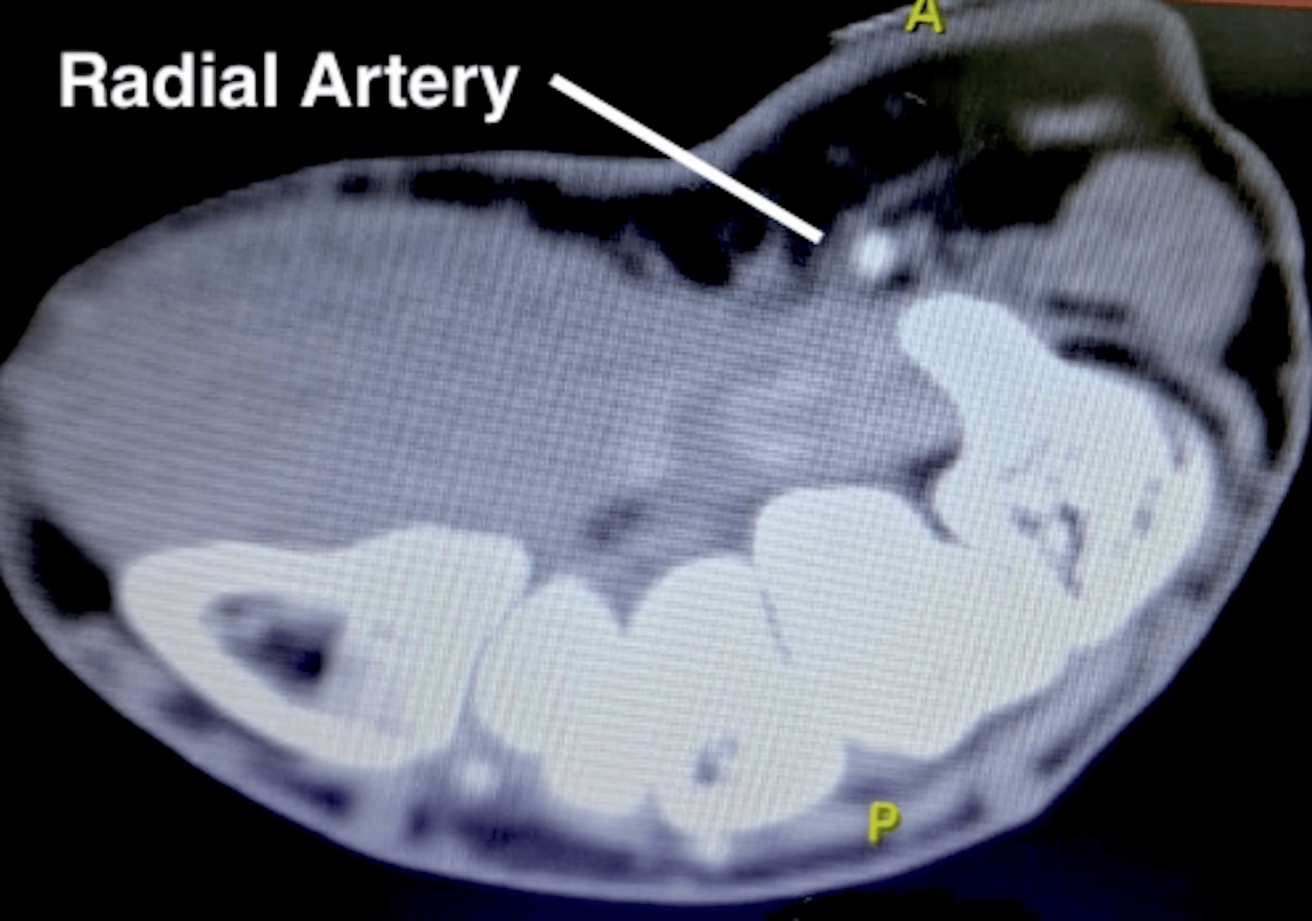
CT angiography at wrist This angiographic image, in coronal view at the wrist, shows no contrast spill suggestive of arterial injury. CT, computer tomography

The CT angiography image of the upper limb at the wrist shows no contrast spill, indicating that there was no demonstrable vascular injury (Figure 1). 

**Figure 2:**
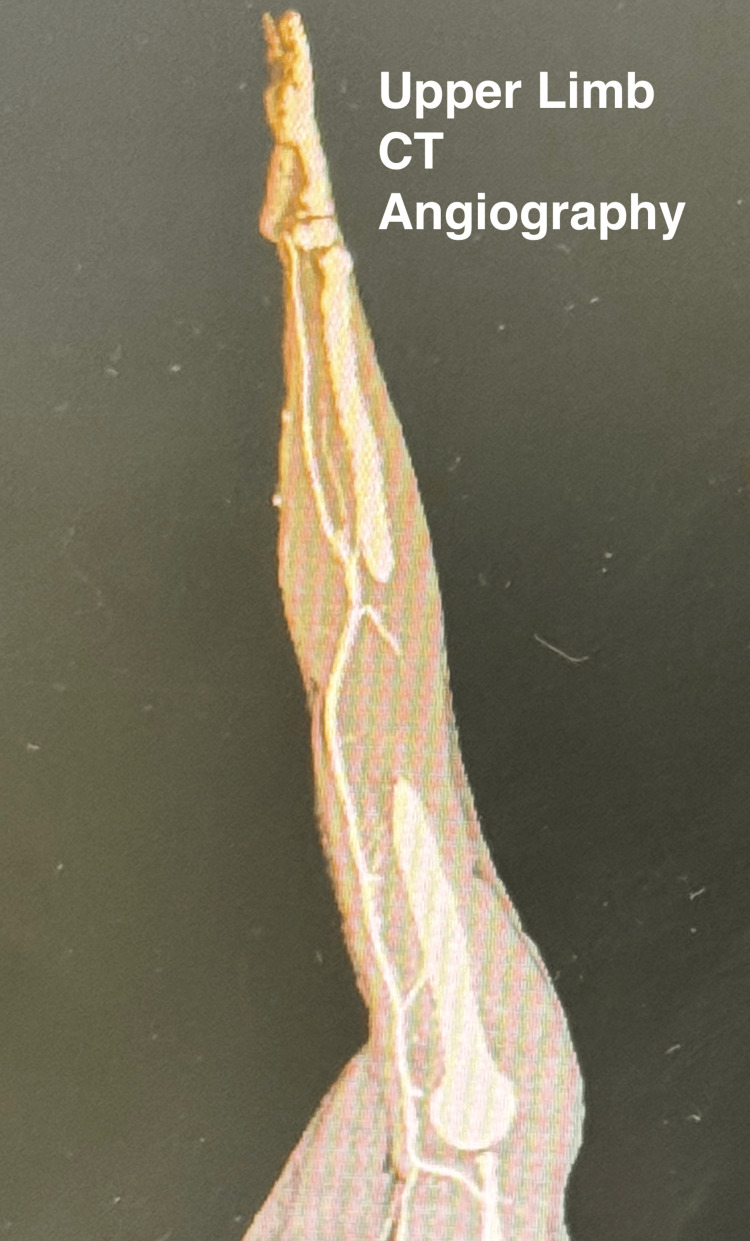
CT angiography of hand CT angiographic view of the hand in sagittal section shows an intact radial artery. CT, computed tomography

Another view of the CT angiography image of the same anatomical area, in sagittal view, also did not show any arterial tear (Figure 2). 

**Figure 3:**
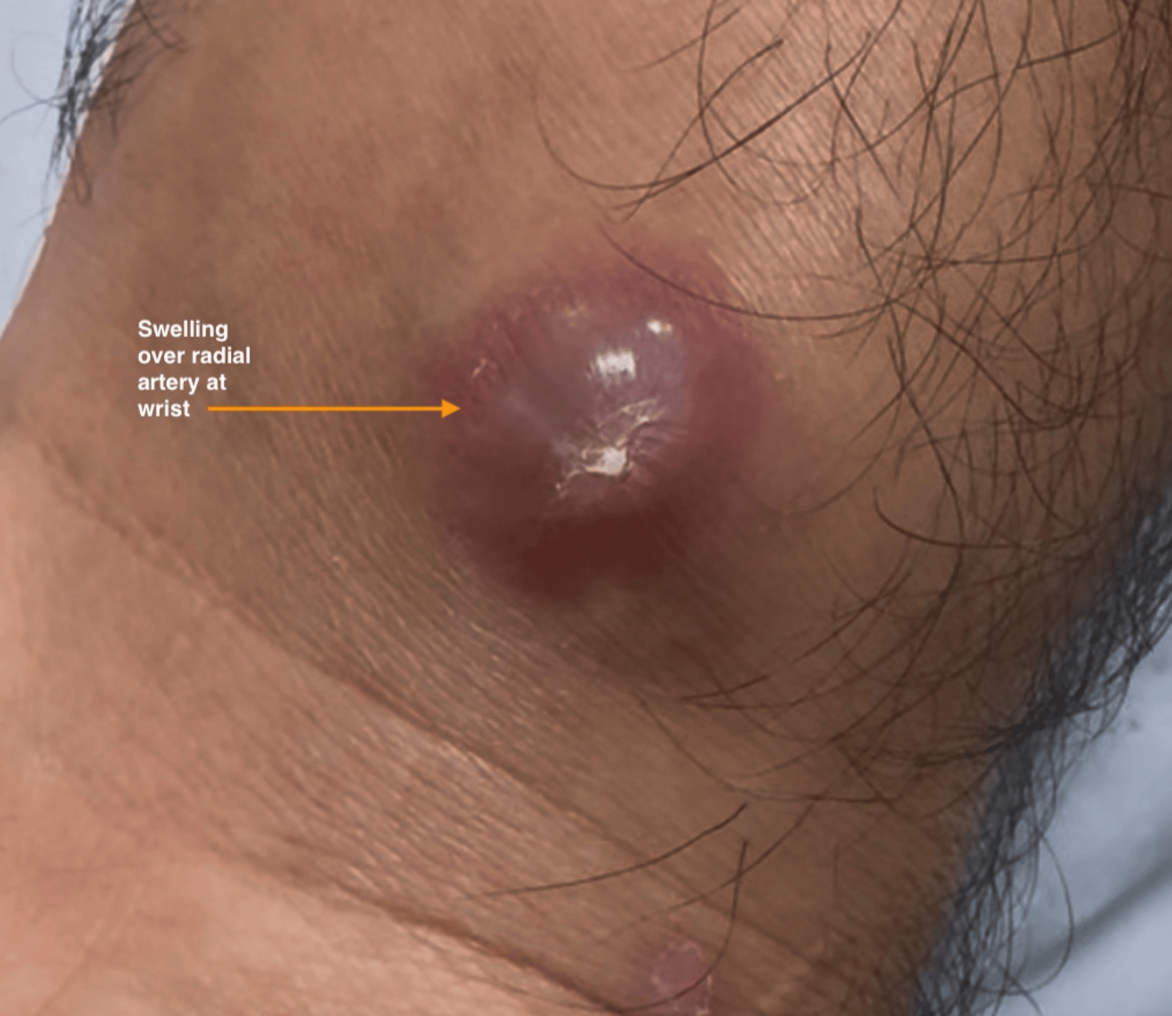
Anterior left wrist area This clinical image shows a pink, pulsatile swelling, approximately 1 × 1.2 cm, on the anterior aspect of the left wrist, directly over the radial artery, at the previous site of injury.

The wound was closed with a single non-absorbable suture, and the patient was discharged. Ten days later, the patient returned with a pulsatile swelling at the site of the previous laceration, raising clinical suspicion for a pseudoaneurysm. The front of the wrist shows a pinkish, soft, and pulsatile swelling (Figure 3). Hand examination was otherwise unremarkable. 

**Figure 4:**
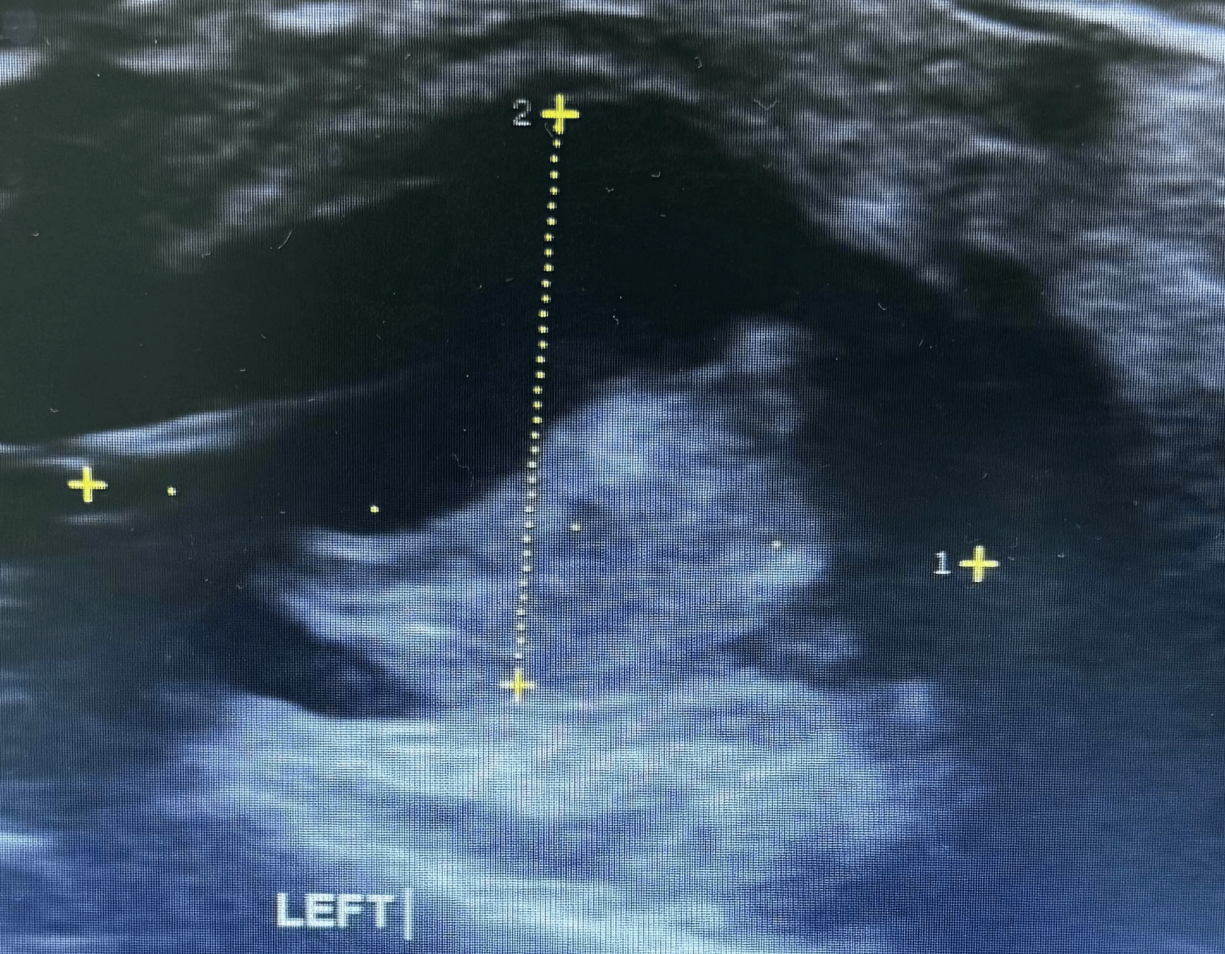
POCUS image of the left radial artery This bedside ultrasound image of the left radial artery, at the site of the aneurysm, clearly shows a flap within the lumen of the artery and a compromised diameter. POCUS: point-of-care ultrasound

Doppler ultrasonography subsequently confirmed a radial artery pseudoaneurysm with evidence of partial thrombus formation, necessitating further intervention. The following ultrasound image of the aneurysmal swelling at the wrist shows a flap within a clearly compromised lumen of the artery (Figure 4).

**Figure 5:**
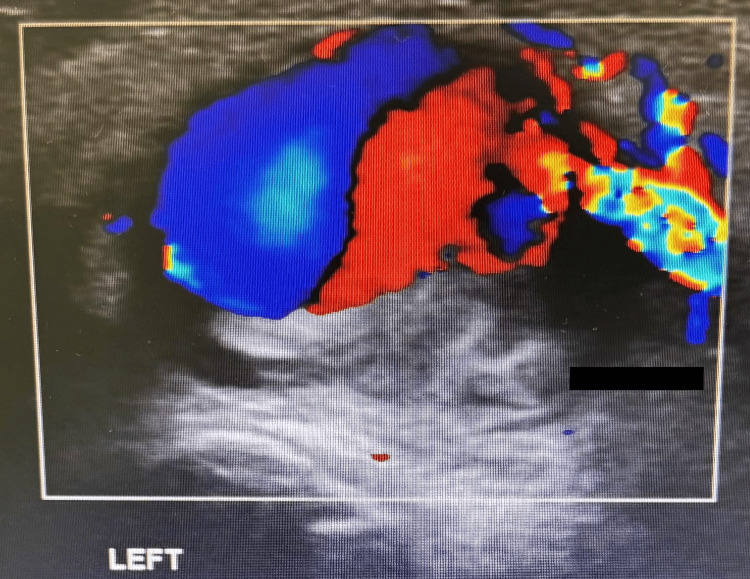
Color Doppler image of the radial artery Color Doppler of the left radial artery at the wrist clearly shows a divided lumen, with spill into the aneurysm.

A subsequent Doppler ultrasound scan of the artery, performed by a specialist radiologist, depicts a divided lumen and demonstrates aneurysm formation (Figure 5). 

The patient was then referred to a vascular surgeon and underwent operative repair of the radial artery. Allen's test, performed pre- and post-arterial repair, was negative, indicating good radial as well as ulnar circulation distal to the injury. He underwent excision and end-to-end anastomosis of the radial artery. His postoperative recovery was uneventful.

## Discussion

This case demonstrates that even minor lacerations can result in delayed vascular complications. A pseudoaneurysm occurs when a defect in the arterial wall allows blood to collect within surrounding tissues, with persistent communication to the parent vessel. Over time, these can enlarge, thrombose, or rupture, and may lead to ischemic complications. The atypical presentation of a pseudoaneurysm developing from a minor laceration, rather than the more common iatrogenic causes, underscores the importance of thorough initial assessment and vigilant follow-up [5,6].

While CT angiography is a sensitive modality for evaluating vascular trauma, small partial-thickness injuries may not be detected initially. In such cases, bedside point-of-care ultrasound (POCUS) can serve as a valuable adjunct, offering rapid, non-invasive evaluation. Previous studies have highlighted the importance of training ED physicians in basic vascular ultrasound to aid in the early diagnosis of complications. This case further emphasizes that even seemingly benign injuries, like those sustained from minor trauma, warrant a high index of suspicion for underlying vascular compromise, especially when initial imaging may not fully capture the extent of the damage [7,8]. The increasing prevalence of vascular trauma, particularly from penetrating injuries and iatrogenic causes, necessitates comprehensive diagnostic strategies to mitigate potential morbidity [9,10].

Minor-appearing lacerations involving vascular structures can lead to serious complications, such as pseudoaneurysm. Careful evaluation, documentation, and the use of bedside ultrasound can reduce morbidity. This case report highlights how vascular injuries can be deceptive and complicate healing, emphasizing the need for heightened clinical suspicion beyond typical iatrogenic etiologies [11]. While iatrogenic causes, particularly those following arterial puncture for radiological or cardiac endovascular procedures, account for a significant proportion of pseudoaneurysms, this case highlights that traumatic lacerations - even minor ones - can also result in such formations [12]. Therefore, vigilance for vascular injury signs, both hard and soft, is crucial for timely diagnosis and intervention [13]. Prompt recognition of arterial injuries, whether from blunt or penetrating trauma, is critical, as delayed diagnosis can lead to significant morbidity, including limb loss [14].

## Conclusions

An adult patient presented with a minor wrist injury, initially appearing trivial in nature. Despite the use of advanced imaging modalities, such as an ultrasound Doppler scan and CT angiography, no vascular injury was detected at the time of the first assessment. However, the patient later developed a radial artery aneurysm, highlighting the potential for delayed vascular complications, even after apparently acceptable initial investigations. This case underscores the critical importance of thorough evaluation, vigilant follow-up, and careful management in patients with wrist injuries, as early recognition and intervention are essential to prevent significant morbidity. We hope this case will help our fellow colleagues in emergency, surgical, and trauma specialties, and provide insight into unexpected outcomes of even seemingly minor injuries.
